# Atypical Porcine Pestivirus Circulation and Molecular Evolution within an Affected Swine Herd

**DOI:** 10.3390/v12101080

**Published:** 2020-09-25

**Authors:** Alba Folgueiras-González, Robin van den Braak, Bartjan Simmelink, Martin Deijs, Lia van der Hoek, Ad de Groof

**Affiliations:** 1 Department Discovery & Technology, MSD Animal Health, Wim de Körverstraat 35, P.O. Box 31, 5830AA Boxmeer, The Netherlands; alba.folgueiras.gonzalez@merck.com (A.F.-G.); robin.braak.van.den@merck.com (R.v.d.B.); bartjan.simmelink@merck.com (B.S.); 2 Laboratory of Experimental Virology, Department of Medical Microbiology, Amsterdam UMC Location AMC, University of Amsterdam, Meibergdreef 9, 1105AZ Amsterdam, The Netherlands; m.deijs@amsterdamumc.nl (M.D.); c.m.vanderhoek@amsterdamumc.nl (L.v.d.H.)

**Keywords:** pestivirus, atypical porcine pestivirus (APPV), viral persistence, congenital tremor, swine, asymptomatic, genomic sequence, phylogenetic analysis, purifying selection

## Abstract

Atypical porcine pestivirus (APPV) is a single-stranded RNA virus from the family Flaviviridae, which is linked to congenital tremor (CT) type A-II in newborn piglets. Here, we retrospectively investigated the molecular evolution of APPV on an affected herd between 2013 and 2019. Monitoring was done at regular intervals, and the same genotype of APPV was found during the entire study period, suggesting no introductions from outside the farm. The nucleotide substitutions over time did not show substantial amino acid variation in the structural glycoproteins. Furthermore, the evolution of the virus showed mainly purifying selection, and no positive selection. The limited pressure on the virus to change at immune-dominant regions suggested that the immune pressure at the farm might be low. In conclusion, farms can have circulation of APPV for years, and massive testing and removal of infected animals are not sufficient to clear the virus from affected farms.

## 1. Introduction

The genus Pestivirus, belonging to the Flaviviridae family, includes single-stranded, positive-sense RNA viruses of veterinary importance, causing economically relevant diseases in livestock animals, but also affecting wildlife species [1]. While for decades the International Committee on Taxonomy of Viruses (ICTV) only recognized the four so-called classical pestiviruses, namely bovine viral diarrhea virus types 1 and 2 (BVDV-1 and BVDV-2), classical swine fever virus (CSFV) and border disease virus (BDV), the discovery of seven novel atypical pestiviruses in the last few years led to the proposal of a new species-independent classification from Pestivirus A to Pestivirus K. Thus, atypical porcine pestivirus (APPV) is now classified as Pestivirus K [2]. Moreover, new pestivirus genomes in bats [3] and piglets (LINDA virus [4]) have been discovered lately, but not yet classified.

Congenital tremor (CT) in newborn piglets, also known as myoclonia congenita, “shaker pigs” or “dancing pigs”, was first reported in 1922 [5]. Its characteristic clinical signs involve tremors of the head and limbs which worsen on stress situations, but are almost gone during sleep. Even though most piglets are clinically healthy at weaning time, the earlier shivering hinders their ability to feed normally from their mother, thus increasing the risk for inadequate colostrum intake, growth retardation or even death by starvation [6]. CT is divided based on its pathology into type A, which displays morphological lesions on the central nervous system, and type B, which does not show any visible morphological lesion [7]. Regarding the distinct causes of disease, type A was further differentiated into five subtypes. Type A-I is associated with one of the classical pestiviruses, CSFV, and characterized by cerebellar hypoplasia in affected pigs [8]. Types A-III and A-IV are both related to different genetic defects only present in the Landrace breed and Saddleback breed, respectively [9,10]. Type-V, also defined by cerebellar hypoplasia, is caused by food poisoning by trichlorfon in pregnant sows [11]. Meanwhile, during the decades, the causing agent of CT type A-II remained unknown, although an infectious agent was suspected [12].

APPV was first characterized in 2015 by Hause et al. [13] through metagenomic sequencing performed on a sample positive for porcine reproductive and respiratory syndrome virus (PRRSV). After its discovery, two independent studies proved APPV’s link to congenital tremor in newborn piglets via the inoculation of pregnant sows with infectious animal material [14,15]. However, Koch’s postulates have not been fulfilled yet due to the difficulties in establishing an appropriate substrate for in vitro culture and retrieval of pure virus stocks [16]. In 2017, Lamb et al. [4] reported a novel pestivirus, named LINDA virus (lateral-shaking inducing neurodegenerative agent), in an Austrian cohort of CT piglets. Although the clinical manifestations and pathology were similar to the ones of APPV, further phylogenetic analysis showed its proximity to Bungowannah virus, a pestivirus never detected outside Australia.

A research study by Postel et al. [17] analyzed 1460 samples of apparently healthy pigs from different countries. They showed that the prevalence of APPV genotypes was around 10% and up to 60% of the tested animals were seropositive. Kaufmann et al. [18] showed that APPV has been circulating in Switzerland at least since 1986, which was the earliest detection point worldwide. To date, APPV has been reported on four continents and fourteen different countries [19]. Mortality is high in CT-affected litters, from around 10% to as high as 30% in some affected farms [14,15,16].

APPV is a highly variable enveloped virus which contains a linear, non-segmented, positive-sense RNA strand of around 11–12 kb. A single open-reading frame (ORF) encodes a single polyprotein of 3635 amino acids on average, which is assumed to be cleaved co- and post-translationally into four structural (the core protein C and the envelope glycoproteins Erns, E1 and E2) and eight non-structural proteins (Npro, p7, NS2, NS3, NS4A, NS4B, NS5A and NS5B) [13]. Several novel APPV sequences have been described since the first report in 2015, further dividing the phylogenetic tree and proposing distinct divisions into three, four or five different genotypes and into numerous particular subclades [20]. To date, 58 unique full-genome sequences are available in NCBI GenBank (access date: 1 April 2020). The APPV full-genome sequences have 80–99% identity, showing high variability even within the same country, with difficulty to infer the origin, dissemination and common ancestor of several strains of the virus [19,21].

High variability in RNA viruses, caused by the lack of proofreading repair mechanisms in RNA polymerases, provides them with a selective advantage, promoting rapid adaptation to novel hosts and environments [22]. On the other hand, excessive error rates can also lead to lethal mutagenesis and threaten the viability of the viral populations [23]. Therefore, hypervariable regions that are thought to contribute to immune escape and adaptation, as well as hyperconserved elements that are fundamental for virus replication and maintenance in the population are main research topics for molecular evolution studies [24].

In the current study, we retrospectively investigated the molecular evolution and functional implication of nucleotide and amino acid substitutions in the APPV genome in a farm in the North Brabant province of The Netherlands over a period of seven years. Sampling was done consistently over time in the controlled environment of a closed-herd farm, where the full farming process is carried out within the farm, and the breeding stock is replaced by gilts from the farm itself. Therefore, by eliminating the input of animals from outside, the risk of novel viral introductions is minimized. In 2013, a first CT outbreak with positive cases of APPV was reported in the farm. Two years later, in 2015, a second APPV outbreak occurred in the same farm with clinically affected, trembling piglets that tested positive. Since then, serum samples were taken from CT piglets at the time of the outbreaks, as well as from apparently healthy pigs as part of regular farm monitoring for APPV and other porcine viruses. Pigs that tested positive were removed from the breeding population to stop the spread of the virus via persistently infected animals. However, several re-emerging peaks of viremic animals were been detected in the farm during the monitoring period, suggesting the continued circulation of APPV in clinically healthy animals.

To assess the possible biological significance of the selective pressures that shape the evolution of APPV within the farm, we looked for evidence of purifying and diversifying selection in the APPV genome. Although viral surface glycoproteins like E2 are well-known targets of positive selection, and variations in these regions can help in immune escape, we also took a full-genome study approach to get a more general, helicopter view and investigate other regions that might have been underestimated until now. The correlation of clinical CT outbreaks with fixation events of non-synonymous, but also synonymous, substitutions can reveal the link to pathogenicity and infectivity of the virus. Here, we reported, to our knowledge, the first longitudinal study on the evolution of the full-length APPV genome within a controlled farm environment. We compared molecular evolution and variability in the genome sequences found on the farm with full-genome sequences from different locations around the world.

## 2. Materials and Methods

### 2.1. Sample Collection

In 2013, the farm under study increased the population of breeding sows from 280 to 570 sows and relocated to new facilities. Piglets stayed in the farrowing unit after weaning (i.e., litters were not mixed). Piglets were vaccinated with a porcine reproductive and respiratory syndrome virus (PRRSV) live vaccine at 2 weeks of age, and with a combined porcine circovirus type 2 (PCV2) and *Mycoplasma hyopneumoniae* (*M. hyo*) vaccine at 3 weeks of age. PRRSV was regularly diagnosed in the farm and therefore a vaccination strategy in sows and piglets was set up. There were no other pathogens diagnosed in regular screenings.

Serum and fecal samples were obtained from gilts and congenitally trembling piglets during the CT outbreaks in 2013 and 2015. Between January 2016 and April 2016, serum was collected from all gilts selected for breeding (196 animals) at the age of first insemination for APPV monitoring and removal of positive animals.

To monitor the presence of APPV in serum over time, five litters with CT born from gilts during the 2015/2016 outbreak were repeatedly sampled until the age of 18 weeks. Moreover, 6 gilts (4 APPV-positive at 5 and 18 weeks of age, and 2 negative) and 2 boars (1 APPV-positive at 5 and 18 weeks of age, and 1 negative), were followed for APPV presence in serum and fecal shed until the age of 10 months. Two APPV-positive gilts were co-housed with one negative gilt, and the boars were housed next to each other with direct contact.

From April 2016 to 2019, clinically healthy 10-week-old gilts in the sow breeding line maintained on the farm were regularly monitored for the presence of APPV in serum. Samples from animals with congenital tremor symptoms were also taken for analysis. In total, 1498 samples were taken during this period.

Blood was collected using the Vacuette 5/8 mL Sep Clot Activator (Greiner-Bio One, Kremsmünster, Austria) and serum was obtained by centrifugation for 10 min at 3200× *g* at 4 °C. Fecal samples were collected using Sigma Virocult swabs and vials (MWE, Corsham, UK), vortexed, transferred to 1.5 mL Eppendorf tubes and centrifuged for 10 min at 10,000× *g* at 4 °C.

RNA was extracted from 200 µL samples by the automated MagNA Pure 96 system (Roche Applied Science, Manheim, Germany) using the protocol ‘Viral NA plasma external lysis SV3.1′.

### 2.2. Quantitative Reverse Transcription

A universal, quantitative, reverse transcription PCR (qRT-PCR) was used to quantitatively detect APPV in samples using primers in the 5′-untranslated region (UTR) of the genome (APPV-PAN2-F3-B: CGYGCCCAAAGAGAAATCGG and APPV-PAN2-R3-B: CCGGCACTCTATCAAGCAGT) [14]. One-step qPCR reactions were performed in a final volume of 50 µL containing 1 µL SuperScript III RT/Platinum Taq Mix (Thermo Fisher Scientific, Waltham, MA, USA), 25 µL 2× SYBR Green Reaction Mix (Thermo Fisher Scientific, Waltham, MA, USA), 17 µL water, 1 µL forward primer (10 µM), 1 µL reverse primer (10 µM) and 5 µL of the RNA isolate. Thermocycling was performed in the CFX96 Touch real-time PCR detection system (Bio-Rad Laboratories, Hercules, CA, USA) starting with an RT reaction for 3 min at 55 °C, a pre-denaturation step for 5 min at 95 °C and 40 cycles of 15 s at 95 °C (denaturation) and 30 s at 60 °C (annealing and elongation). The specificity of SYBR Green qPCR was validated by melting curve analysis between 65 °C and 95 °C with an increasing gradient of 0.5 °C per 5 s. Results were analyzed with the CFX Manager software (Bio-Rad Laboratories, Hercules, CA, USA).

To obtain a standard curve for the quantitative analysis, a recombinant bacterial plasmid containing the 5′-UTR PCR target was made (GenScript, Piscataway, NJ, USA). The copy number of the recombinant plasmid was calculated, and eight dilution series (10^8^–10^1^ copies/µL) were included in duplicate in RT-qPCR to calculate the number of virus copies per µL.

### 2.3. Viral Genome Amplification and Sanger Sequencing

A starting sequence of 1073 bp obtained from a previous study using Illumina Sequencing (Amsterdam UMC, Amsterdam, The Netherlands) was used as reference [14]. This short read was mapped to the 58 full-genome sequences available in NCBI GenBank (access date: 1 April 2020). The three most similar full-genome sequences (GenBank accession numbers: KY624591.1, MH885413.1 and KX778724.1) were aligned by CLUSTALW using Geneious Prime v2019.0.4 (http://www.geneious.com) (Geneious, Auckland, New Zealand), and the consensus sequence was extracted for primer design.

The cDNA from the extracted RNA of the clinical samples (Section 2.1) was synthesized using the QuantiTect reverse transcription kit (Qiagen, Hilden, Germany) following the manufacturer’s manual. The entire viral genome from the 2013 sample was amplified using an initial series of twelve overlapping PCRs. Gaps and faulty PCRs were solved following a genome walking strategy (amplification and sequencing primers available in Supplementary Table S1). PCR reactions were performed in a final volume of 25 µL containing 12.5 µL 2× Phusion High-Fidelity PCR Master Mix with HF Buffer (New England BioLabs, Ipswich, MA, USA), 0.75 µL DMSO 100%, 6.75 µL water, 1.25 µL FW 10 µM primer, 1.25 µL REV 10 µM primer and 2.5 µL of template cDNA. Thermocycling was performed in the CFX96 Touch real-time PCR detection system (Bio-Rad Laboratories, Hercules, CA, USA) using an initial denaturation step at 98 °C for 5 min, 40 cycles of 30 s at 98 °C (denaturation), 30 s at the annealing temperature optimal for the primer set and 1 min and 30 s at 72 °C (elongation), followed by 7 min at 72 °C. The PCR products were stored at 4 °C until further analysis. Five microliters of the PCR products were analyzed by agarose gel electrophoresis (1.4% *w*/*v* agarose) to check the fragment size and the specificity of the amplification. PCR products were purified using the QiaQuick PCR purification kit (Qiagen, Hilden, Germany) following the manufacturer’s manual.

Sequencing PCR reactions were performed using the BigDye Terminator v3.1 cycle sequencing kit (Applied Biosystems, Carlsbad, CA, USA) in a final volume of 20 µL containing 4 µL BigDye Terminator ready reaction mix, 3 µL 2× Phusion High-Fidelity PCR Master Mix with HF Buffer (New England BioLabs, Ipswich, MA, USA), 0.5 µL DMSO 100%, 2.5 µL WFI, 2.5 µL FW or REV 10 µM primer and 7.5 µL of the purified PCR product. Thermocycling was performed in 30 cycles of 10 s at 95 °C (denaturation), 10 s at the annealing temperature optimal for the primer set and 2 min at 60 °C (elongation). The PCR products were stored at 4 °C for further analysis. The cycle sequencing products were purified using the DyeEx 2.0 Spin kit (Qiagen, Hilden, Germany) following the manufacturer’s manual. Capillary electrophoresis was performed using the 3500 Genetic Analyzer (Applied Biosystems, Carlsbad, CA, USA) and data were analyzed using the Sequencher 5.4.6 (Gene Codes, Ann Arbor, MI, USA) and Geneious Prime v2019.0.4 (Biomatters Ltd., Auckland, New Zealand) software.

Based on the obtained sequence, Sanger sequencing was performed following the same methodology on the other five samples (years 2015, 2016, 2017, 2018 and 2019) by GenScript (Piscataway, NJ, USA).

### 2.4. Determination of E2 Sequences

The amplification of E2-coding sequences was performed using a two-step RT-PCR protocol as described in Section 2.3. The amplification and sequencing PCR reactions of 857 bp and 953 bp fragments were performed using two primer pairs flanking the E2-coding region (E2-F1: 5′-TGGTGCCTATTGTTGTCAGG-3′, E2-R1: 5′-AGTTCTTCCTTGACGGCTAG-3′, E2-F2: 5′-GCCCTGGTGAACATAGTCAC-3′ and E2-R2: 5′-TCCTTGACGGCTAGCATTATG-3′). Capillary electrophoresis was performed using the 3500 Genetic Analyzer (Applied Biosystems, Carlsbad, CA, USA) and trimmed sequences (702 bp) were analyzed using Geneious Prime v2019.0.4 (Biomatters Ltd., Auckland, New Zealand).

### 2.5. Submission of Sequences

The sequences of the APPV genomes were deposited in GenBank under the accession numbers MT512531–MT512537. The partial E2-coding sequences were deposited in GenBank under the accession numbers MW011356–MW011406.

### 2.6. Nucleotide and Amino Acid Analysis of Variants

The obtained partial E2-coding sequences were aligned using the CLUSTALW translation alignment in Geneious Prime v2019.0.4 (Biomatters Ltd., Auckland, New Zealand) with BLOSUM cost matrix, a gap open cost of 5 and a gap extended cost of 4.

The fifty-eight full-genome APPV sequences from NCBI GenBank database (access date: 1 April 2020) were aligned along with the sequences obtained in the current study [25]. Multiple sequence alignment was performed using the CLUSTALW translation alignment in Geneious Prime v2019.0.4 (Biomatters Ltd., Auckland, New Zealand) with BLOSUM cost matrix, a gap open cost of 10 and a gap extended cost of 6.66. For the farm dataset, we called single nucleotide substitutions on the aligned genomes using Geneious Prime v2019.0.4 and differentiated them into synonymous and non-synonymous. Ambiguous nucleotide and amino acid calls, which are considered by the software as variants, were treated as missing data.

The prediction of *O*-glycosylation and *N*-glycosylation motifs in the Erns, E1 and E2 glycoproteins were performed with DictyOGlyc1.1, NetOGlyc4.0 and NetNGlyc1.0 prediction algorithms via the webserver from the Technical University of Denmark Department of Bio and Health Informatics (DTU Bioinformatics, http://www.cbs.dtu.dk/services/) [26,27].

### 2.7. Phylogenetic Analysis and Estimation of Evolutionary Rates within the Farm

The Recombination Detection Program version 4 (RDP4) was used to screen for recombination events on the multiple sequence alignment of the six APPV genomes found in the farm, using RDP, GENECONV, BootScan, Maxchi, Chimaera, Siscan and 3Seq methods. Recombination was considered when *p*-value was <0.0001 and the recombinant score was >0.6 [28].

The full-genome APPV sequences available in NCBI and the six APPV in-farm sequences obtained in this study were aligned using the CLUSTALW translation alignment in Geneious Prime v2019.0.4 (Biomatters Ltd., Auckland, New Zealand) with BLOSUM cost matrix, a gap open cost of 10 and a gap extended cost of 6.66. The phylogenetic tree was created with the MEGA X software using the neighbor-joining method with the Kimura two-parameter substitution model [29,30]. Complete deletion was done in case of gaps or missing data. The analysis was performed for 500 bootstrap replicates. An estimation of the evolutionary rates was calculated for the obtained E2-coding sequences, as well as for the six full-genome sequences using the TempEst v1.5.3 software [31].

### 2.8. Selection Pressure Analysis

Selection pressure analyses were done for the multiple sequence alignment of the six farm sequences as well as for the multiple sequence alignment including the NCBI full-genome APPV sequences. Selection pressure analyses were performed on the full ORF, using mixed-effects model of evolution (MEME) and fixed-effects likelihood (FEL). All these algorithms were implemented on the HyPhy (Hypothesis Testing using Phylogenies) open-source software package and can be accessed through the Datamonkey webserver (https://www.datamonkey.org/) [32].

MEME uses a mixed-effect maximum likelihood approach. The algorithm estimates a synonymous α parameter (d_S_) and a two-category mixture of non-synonymous (d_N_) β parameters for each site. MEME infers two ω (d_N_/d_S_) classes and uses a likelihood ratio test to compare between the models and check for episodic diversifying selection. A significance threshold of *p* < 0.1 was used on the analysis [33].

The fixed-effects likelihood (FEL) algorithm was used in order to detect negatively selected sites on the alignment. FEL uses a maximum likelihood approach to infer non-synonymous (d_N_) and synonymous (d_S_) substitution rates, also assuming a constant selection pressure for each site on the alignment. All branches were tested for selection. A model with synonymous rate variation, where the d_S_ parameter in the codon model is allowed to vary across sites, was used for the analysis. A significance threshold of *p* < 0.1 was used on the analysis [24].

## 3. Results

### 3.1. 2013–2016: Congenital Tremor Outbreaks, Follow Up Studies and Eradication Strategy Design

In 2013, the farm increased the internal population of breeding sows, aimed to breed gilts for the replacement of production sows (sows breeding line) from 280 to 570 sows and, at the same time, relocated to new facilities with state-of-the-art climate control and housing conditions. Piglets stayed in the farrowing unit after weaning, thus litters were not mixed. In the same year, a first large-scale CT outbreak was reported on the farm and a second large-scale outbreak occurred at the end of 2015/early 2016. Both outbreaks were related with positive cases of APPV and various other clinical observations (e.g., reduced vitality and mortality of the piglets, and increased return to estrus percentage). In the time between 2013 and 2015, no major abnormalities were observed, although incidentally few piglets with tremors were seen, but with no impact on production.

During the 2015–2016 outbreak, CT prevalence varied from 5% in litters from fourth parity sows, up to 55% in litters from first and second parity sows. In the latter case, the mortality in the farrowing unit ranged from 25% in litters from second parity sows to 69% in the ones from first parity sows. The related effects on the production data are shown in Supplementary Table S2.

After the second large-scale outbreak, it was decided to monitor all gilts selected for breeding for the presence of APPV, both purebred line gilts and production gilts. Serum of those animals was analyzed at the age of first insemination with the aim to remove positive gilts from the breeding population. A universal, quantitative, reverse transcription PCR (qRT-PCR) was used to quantitatively detect APPV on pig serum samples. This strategy was applied between January and April 2016, during which time 196 gilts were tested and 15% of them tested positive for APPV (Supplementary Table S3).

During parallel monitoring of pigs born with CT and positive for APPV in serum, we observed that a significant percentage of pigs turned PCR-negative for APPV in serum around the age of 18 weeks. The piglets born with CT were considered as persistent carriers of the virus and, at the time of weaning, the virus was still present in the piglets that still showed recognizable, but less severe, tremors. A follow-up of these piglets showed that the percentage of APPV-positive PCR scores in serum was reduced to 45% at 18 weeks of age, with viral copy numbers in serum also being greatly reduced (Supplementary Table S4).

To gain further insight into the dynamics of APPV viremia, 6 gilts (4 APPV-positive at 5 and 18 weeks of age, and 2 negative), and 2 boars (1 APPV-positive at 5 and 18 weeks of age, and 1 negative), were followed for APPV presence in serum until the age of 10 months. Two APPV-positive gilts were co-housed with one negative gilt, in separate cages, and the boars were housed next to each other with direct contact. No APPV was detected in any of the 8 animals at the age of 24 weeks and at any time point thereafter, with the exception of 1 boar testing PCR-positive at the age of 32 weeks (Supplementary Table S5). Fecal shed from the same animals was also monitored until the age of 10 months via qPCR. Results showed, in the age range between 24 and 44 weeks, intermittent shedding in the feces of the persistently infected (PI) gilts and temporary presence in the feces of horizontally infected gilts (Supplementary Table S6).

### 3.2. 2016–2019: Re-Emergence of Congenital Tremor and Monthly Quantitative Detection of APPV

Since April 2016, when regular sampling from clinically healthy 10-week-old gilts in the sow breeding line maintained on the farm started, serum samples were received monthly for APPV monitoring purposes. Until December 2019, a total of 1505 serum samples from 10–16-week-old pigs were analyzed.

**Figure 1:**
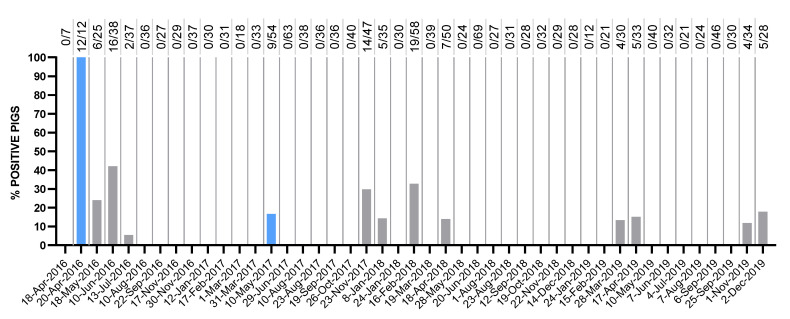
Percentage of atypical porcine pestivirus (APPV)-positive pigs detected during monthly screening in the farm by RT-qPCR analysis. The *x*-axis shows the sampling date from April 2016 to December 2019. The percentage of positive samples is shown in the *y*-axis. Blue-colored columns show that positive PCR results were accompanied by clinical CT symptoms on those specific months—i.e., April 2016 and May 2017. The number of PCR-positive animals in relation to the total number tested each specific month is shown above the columns.

Figure 1 shows the percentage of APPV-positive animals among the tested set for each month since the screening started in April 2016 until the last analyzed data from December 2019. In this period, two peaks of CT symptoms in newborn piglets were detected in the farm in April 2016 and May 2017 (blue columns in Figure 1). In order to diminish horizontal transmission of the virus in the sow breeding population, infected APPV pigs detected during the monitoring were removed from the population as these were likely persistently infected.

### 3.3. Characterization of APPV Sequences

Forty-eight E2-coding sequences were determined from the APPV-positive samples collected during monthly monitoring in the farm at different time points between 2016 and 2019, along with three sequences obtained previously in 2013 and 2015 during the large-scale CT outbreaks (Supplementary Table S7). The E2-coding sequences showed a maximum pairwise genetic distance of only 0.71%. The low genetic diversity observed within the farm supported the hypothesis that only one viral strain was circulating in the herd with no viral introductions from the outside.

The amino acid sequences were also compared according to the collection date (Supplementary Figure S1). Samples obtained from different animals at the same time point showed a completely identical genome, with the exception of the highly mutated codon D752, varying to either Gly, Ser or Asn residues (Section 3.5). Besides, a more thorough approach was taken for the samples obtained from affected animals at the time of the CT outbreaks, with 8 out of 12 sequences obtained in April 2016 and 8 out of 9 in May 2017. No characteristic differences were seen at those time points that could lead to an explanation of the distinct symptoms in relation to the nucleotide or amino acid sequences.

### 3.4. Phylogenetic Analysis and Estimation of Evolutionary Rates of APPV within the Farm

**Figure 2:**
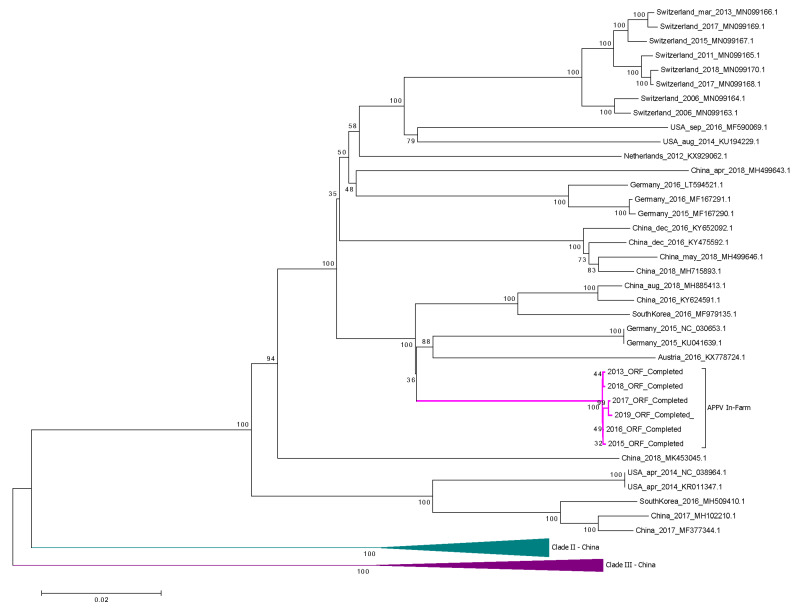
Phylogenetic analysis of the complete coding region alignment of atypical porcine pestivirus (APPV) sequences worldwide obtained from NCBI database and six APPV sequences retrieved in the present longitudinal study on the Dutch swine farm between 2013 and 2019. The neighbor-joining method with the Kimura two-parameter substitution model was used. Gaps and missing data were subjected to complete deletion. Bootstrap values are provided at the root of the clusters. The scale bar is a measure of the proportion of divergence. Run for 500 bootstrap replicates. Clade I shown as expanded tree. Clades II and III, containing only sequences from China, are collapsed and shown in green and purple in the lower part of the tree. APPV branches obtained in the current study are shown in pink.

A phylogenetic analysis of the full-length APPV sequences available worldwide and obtained from the NCBI database and six APPV sequences obtained in the present study (Section 3.5) confirmed the division of APPV into three different clades, two of them containing only sequences from China (Figure 2). APPV sequences from the current study clustered in one subtree together with two German sequences (NC_030653 and KU041639, from 2015), one Austrian sequence (KX778724, from 2016), two Chinese sequences (MH885413, from 2018, and KY624591, from 2016) and one South Korean sequence (MF979135, from 2016). The other full-APPV genome from The Netherlands available in NCBI was clustered within the same clade, but into a different subtree, together with eight sequences from Switzerland and two from the US (Figure 2).

### 3.5. Genome and Protein Variations of APPV within the Farm

The samples with the highest numbers of APPV genome copies were selected from each of the years, and six full-length APPV genomic sequences from samples collected in the farm on 2013 and each year between 2015 and 2019, were determined. The 2013 and 2015 APPV genome sequences were obtained from serum samples from CT-affected piglets at the time of the large-scale outbreaks. The length of the complete APPV in-farm coding sequence (excluding the 5′-UTR region) was 10,908 nt, in line with the previously reported length of the coding region [14]. Multiple sequence alignment was performed on these six sequences using the CLUSTALW translation alignment in Geneious Prime v2019.0.4. The sequences from the different years showed a high similarity at the nucleotide level, between 99.74% and 99.90%. The number of nucleotide differences ranged from only 10 nucleotide substitutions between 2017 and 2019 genomes to 26 nucleotide substitutions between 2013 and 2017 genomes. On the amino acid level, the differences ranged from only 1 amino acid substitution between 2017 and 2019 to 7 amino acid substitutions between 2013 and 2017 and between 2016 and 2017 (Table 1).

The nucleotide substitutions found in the alignment were classified regarding their protein location and differentiating synonymous substitutions and non-synonymous substitutions. All substitutions are shown in Supplementary Table S8. Forty-five nucleotide substitutions were found along the APPV genome during the monitoring years in the farm from which thirty-four positions corresponded to synonymous substitutions. Eleven non-synonymous substitutions occurred on the APPV genome in the farm between 2013 and 2019. Among those, one amino acid change, V563A, was in the structural glycoprotein gene E1, while two amino acid changes, I726V and D752G, were in the E2 structural glycoprotein encoding region. The other eight non-synonymous substitutions occurred in the genes of non-structural proteins: two in Npro (S48P and H152L), one in NS2 (R1122K), three in NS5A (K2437R, G2550E and A2824T) and two in NS5B (D2979N and N3166T) (Supplementary Table S8).

Some amino acid changes in the strains have not been described before. The E1 non-synonymous substitution V563A only occurred within the farm, while the genotype of this site worldwide was always conserved as a Val residue. The synonymous substitution found on the same coding gene, V567, was also exclusive, when compared with other sequences in Clade I. Interestingly, site T608 in the in-farm sequences possessed a unique genotype compared with the consensus I608, and the V608 variant present as well in Clades II and III. Moreover, site I790 also showed a unique genotype, found only in one sequence from Switzerland from 2011 (accession number MN099165), while a Val residue is encoded on that position in the sequences worldwide. The glycosylation status of the pestivirus glycoproteins plays an important role in virulence. In this line, the substitution on E2 glycoprotein D752 to a Gly, Ser or Asn residue (Supplementary Table S8, Supplementary Figure S1) modified the 752NDT754 N-glycosylation motif predicted by NetNGlyc1.0 server (http://www.cbs.dtu.dk/services/). In the non-structural proteins, a unique motif was found in the N-terminal protease. The site 126KPAPASR132 was unique within the APPV sequences retrieved from the current farm study, including up to four modifications on its amino acid chain, in comparison with other geographically distinct APPV full genome sequences.

An estimation of the evolutionary rates within the farm was calculated for the full ORF of the APPV genome, as well as for the E2 glycoprotein-the coding sequence using TempEst v1.5.3 software [31]. An evolutionary rate of 3.224 × 10^−4^ substitutions/site/year (correlation coefficient = 0.9009; *R*^2^ = 0.8117) was estimated for the full ORF of APPV over a period of six years of evolution, from 2013 to 2019, within the studied farm. The envelope glycoprotein E2, considered in all pestiviruses as the main antigen eliciting immune response, had an estimated evolutionary rate of 9.347 × 10^−4^ substitutions/site/year (correlation coefficient = 0.7706; *R*^2^ = 0.5938). Both evolutionary rates were in line with the ones reported in other pestivirus species. The evolution of the CSFV E2 gene was estimated between 1.73 × 10^−3^ and 5.76 × 10^−4^ substitutions/site/year [34,35], while the full genome estimation was 1.03 × 10^−4^ substitutions/site/year [36]. For BVDV, evolutionary rates of 1.40 × 10^−4^ substitutions/site/year for the full genome and 1.26 × 10^−3^ substitutions/site/year for the E1-E2 coding region were reported [37]. A recombination analysis was done using the RDP4 software in order to find breakpoints within the multiple sequence alignment that might have undergone recombination, and to screen them for evidence of phylogenetic incongruence previous to phylogenetic tree building [28]. No recombination events were found at the farm level.

### 3.6. Selection Patterns within a Farm: Six-Year Evolution Study on an APPV in-Farm Variant

Negative, also called purifying, selection consists of the evolutionary pressure hindering the fixation of non-beneficial or deleterious protein variations in the population. On the other hand, positive or diversifying selection is the evolutionary pressure that promotes the fixation of beneficial protein variations in the population. This last one can be considered pervasive, when it is constant through time, or episodic, when it is sporadic and affecting only some lineages. Using the algorithms implemented in the HyPhy software packages via the Datamonkey webserver [32], we were able to identify a number of codons potentially subjected to selection pressures on our APPV genome between 2013 and 2019.

No positively selected codons, neither episodic nor pervasive, were found by any of the methods. Thus, positive selection cannot be considered as an evolution pattern within the farm during the six years of the study. On the other hand, FEL (fixed-effects likelihood) found evidence of pervasive negative/purifying selection at 9 sites among the 3635 codons, with a *p*-value threshold of 0.1. The nine codons subjected to purifying selection were found to be in the N-terminal protease (codon 132), the Erns glycoprotein (codon 418), the NS2 protein (codons 1054 and 1198), the NS3 protein (codon 1814), the NS5A protein (codons 2676 and 2848) and the NS5B protein (codons 2905 and 3243). FEL algorithm indicated, under the global MG94xREV model, a total non-synonymous/synonymous rate ratio for the in-farm alignment equal to 0.172, indicating the prevalence of negative selection within the APPV genome sequence.

## 4. Discussion

In 2013, a first large-scale CT outbreak was reported in a farm in The Netherlands, with a second large-scale outbreak at the end of 2015/early 2016, both related to APPV infections. After the second outbreak, all gilts selected for breeding were monitored by serum PCR screening at the age of first insemination for the presence of APPV with the aim of removing infected animals from the breeding population. However, there were some practical limitations to this approach: (1) The removal of a high percentage of the selected gilts at the age of first insemination severely disrupted the normal farm sow replacement strategy. (2) Serological tests were not available at the time and thus, the difference between persistent carriers and gilts with horizontal infections could not be established. (3) Parallel monitoring of persistently infected pigs from the second outbreak revealed that, in contrast to what it may have been expected based on experience with other pestiviruses, APPV disappeared from the serum in adult pigs. The data published by Schwartz et al. [16] showed stable presence of APPV in serum until 14 weeks of age, but our observations on a larger number of animals showed that a significant percentage of pigs turned PCR-negative for APPV around the age of 18 weeks.

Fecal shed was also monitored to potentially identify persistently APPV-infected pigs. In the age range between 24 and 44 weeks, monitoring APPV infection is difficult due to the absence of the virus in serum and intermittent shedding in the feces of PI gilts. Horizontally infected gilts show temporary presence in feces and serum. Therefore, a gilt-monitoring strategy was technically complicated and could lead to false-negative test results and consequently, the maintenance of persistently infected animals in the breeding population. It is of note that APPV-positive fecal shed has not proven to be infectious.

Based on these considerations, we reasoned that the early removal of PI animals in the purebred gilt breeding population, by performing qPCR analysis on serum samples before the age of 16 weeks, was the best strategy to control APPV at the farm level. Hence, a monthly monitoring program for APPV infections in 10-week-old selected breeding gilts of the sow breeding line was set up in the “closed” CT-affected farm with the purpose of reducing the risk of horizontal APPV infections to pregnant gilts and subsequent vertical infection of newborn piglets. It was assumed that these persistent carriers of APPV, often born without showing clinical symptoms after vertical transmission of the virus and with subsequent use in the farm sow replacement breeding program, contributed significantly to the risk of infections of pregnant gilts and low-parity sows in group housing. The results of our analysis showed that even with stringent removal of APPV PCR-positive, assumed persistently infected replacement gilts, the virus cannot be completely eliminated from the farm. The genotype remained unchanged throughout the years, as demonstrated by the low genetic diversity observed in the partial E2-coding sequences from the samples obtained during monthly monitoring, making it unlikely that new introductions via sperm were involved in the continuing APPV infections in the farm. Re-emerging peaks of viremic animals, often without symptoms of the disease but incidentally with the birth of CT piglets during these six years within this closed-herd farm, even with infected breeding animals removed from the population, showed that the virus was difficult to control let alone eliminate. Besides, the E2-coding sequences obtained from the majority of affected animals at the time of the CT outbreaks did not show any characteristic difference at the nucleotide nor amino acid level when compared to the asymptomatic carriers that could lead to an explanation for the clinical manifestation.

The hypervariable regions, which contribute to virus escape from the immune system, as well as the conserved elements involved in crucial virus replication processes and maintenance of the viral population, are obvious targets for drug and vaccine design [24]. Moreover, it can be hypothesized that certain substitutions in the viral coding sequence may be involved in the cyclic peaks of viremia and/or correlate with the pathogenicity of the virus. In the present study, forty-five nucleotide substitutions were found along the APPV genome during the monitoring years in the farm, eleven of them causing an amino acid change. Among those, two occurred on the E2 protein-coding gene, usually recognized as the main antigen able to elicit neutralizing antibodies in infected animals [38,39]. Recent studies on subunit E2 vaccines from APPV have shown strong immune responses in mice [40]. Moreover, E1–E2 heterodimers have been considered in other pestivirus species as key for viral infectivity [38]. Although the results obtained after the analysis of the APPV sequences on the followed farm did not point at any positively selected sites, two of the non-synonymous substitutions found on the heterodimer-coding genes were almost unique to the farm under study. More interestingly, the substitution D752G in E2 glycoprotein, which showed high variability throughout the fifty-one partial E2 sequences analyzed, modified the predicted 752NDT754 *N*-glycosylation motif. Generally, E2 glycosylation sites are highly conserved due to their primary role in viral entry and infection and their removal has shown viral attenuation in other pestivirus species [41]. Further research needs to be done to decipher the functionality of this highly variable motif.

The unique motif 126KPAPASR132 was found in the N-terminal protease in all APPV sequences retrieved from the farm. This motif included up to four modifications on its amino acid chain, in comparison with other geographically distinct APPV full-genome sequences. Although the exact functionality of this site is not known, Npro plays an important role in viral evasion of the innate immune response in other pestivirus species. Npro activity decreases the levels of the interferon regulatory factor 3 (IRF3) via proteasomal degradation, inhibiting its downstream signaling and thus suppressing type-I interferon responses in infected animals [42]. Previous research studies have related the functionality of this protein with the enhancement of co-infections with bovine respiratory syncytial virus (BRSV) in cattle [43]. APPV in persistently infected animals is commonly found together with other viruses and, it may therefore intensify those secondary infections. More importantly, experimental Erns and Npro mutations in the BVDV genome have failed to induce persistent infections in cattle [44].

The other 34 substitutions found along the APPV genome during the six years evolution in the farm, but not leading to amino acid changes, are not less important as the codon usage bias has been related to virus translation efficiency [45], RNA structures critical for replication and packing [46] and enhanced virulence [47]. Future studies on the codon usage and effects on RNA structure using reverse genetics may therefore shed light on the actual effect of synonymous substitutions. We are aware of the fact that, with only six full-genome sequences included in the current study, our results were based on a limited amount of sequence data. However, given the dynamics of the virus at the farm, we had maximized our analysis possibilities. Pigs with high APPV loads in serum, which are needed for a successful sequencing strategy, were only occasionally observed and, in addition, pigs from the same litter were expected to have the same viral genotype, as demonstrated by the partial E2 sequences obtained from a large number of samples, and further sequence analysis would not add meaningful data. Moreover, the limited genome variation shown within the farm suggested that more sequences obtained during the same years would not radically change the observations and conclusions drawn in the present study. Nonetheless, we acknowledge that an increase in the number of APPV full-length sequences available, especially within longitudinal studies, will be key for the scientific community to further understand the evolutionary dynamics and genomic features of this pestivirus.

With regard to the evolutionary pressures of APPV, the present study showed a general genome-wide purifying pressure, especially strong on the non-structural proteins. These results indicated the importance for the virus to maintain the functionality of the non-structural viral proteins, avoiding the fixation of detrimental amino acid substitutions that might hinder the virus ability to evade the host immune system and cause persistent infections. In Flaviviridae, non-structural proteins have been determined as key players in viral escape from the host immune system. Hepatitis C virus (HCV) NS3 protein inhibits tumor necrosis factor alpha (TFN-α) stimulated NF-κB activation to evade the host innate immunity [48]; NS4A proteins of dengue virus 1 (DENV-1) inhibit the interferon- β (IFN-β) signaling pathway mediated by the retinoic acid-inducible gene I (RIG-I) and TANK-binding kinase-1 (TBK1) proteins [49]; and NS5 protein of several flaviviruses is also involved in the inhibition of IFN signaling by degrading the signal transducer and activator of transcription 1 (STAT1) or STAT2 [50,51]. Even though the signaling pathways and target sites of the majority of the non-structural proteins remain unknown for several virus species, these examples acknowledge their potential function in the host innate immune evasion. The strong purifying selection we found could be explained by a lack of pressure from the host immune system, as the persistently infected animals may potentially be immunotolerant, although experimental evidence is needed to ultimately confirm this hypothesis. Regular use of ELISA systems, to monitor whether immunity is raised to the virus, would bring light to this issue [39].

Monitoring studies in APPV-infected farms during the last years showed that APPV caused persistent infections in piglets when they were infected in-utero, occasionally developing congenital tremors, while horizontal infections within the herd were transient and without visible clinical signs [39]. As aforementioned, this is similar to BVDV infections in cattle, which only causes persistent infections in the calves when the virus is transmitted in-utero before 120 days of gestation. While some persistently infected calves are born with congenital malformations, others are clinically healthy with BVDV infections going unnoticed until the onset of clinical signs in the new parities [52]. A widely accepted hypothesis, supported by several research studies, is that non-cytopathic BVDV strains fail to induce type-I interferon responses in the infected fetus developing persistent infections and immunotolerant calves [52,53]. These persistently infected animals shed the virus continuously during their lifetimes, provoking transient horizontal infections within the herds, but more importantly infecting pregnant cows leading to the birth of persistently infected calves [54].

Our study results of APPV circulation on the affected sow farm showed similarities with the ways that BVDV persists within the cattle populations. Persistently APPV-infected animals remained undetected in a population that was not continuously monitored by PCR because they did not always show congenital tremors. Although our control strategy did not result in full elimination of the virus, at least it resulted in no further large-scale outbreaks since early 2016. Our approach was based on the hypothesis that persistently infected gilts are the main contributors to the spread of the virus and thus, removing those indirectly leads to the prevention of infections of pregnant sows. Nevertheless, the combination with a direct approach based on the elimination of infected pregnant animals could further improve the eradication strategy, as experimental studies have shown that virus infection during early stages of gestation leads to viremia and transplacental transfer.

Research on APPV vaccines is still limited. High strain heterogenicity and persistent infections have been a major bottleneck in the case of BVDV vaccination [55], and for APPV the same problems will be encountered when vaccines are designed and tested. Importantly, effective controlling strategies in closed farm situations should start with intensive testing of all the animals in the herd, followed by removal of positive carriers from the population. Testing should not only be done via RT-qPCR in serum, but also in fecal swabs, as it has been observed that the virus disappears from blood while it can still (intermittently) be present in feces [14]. The strategy presented in this study was not adequate to fully eliminate the virus; APPV kept circulating in the sow population after removal of persistent carriers in the sow breeding herd, suggesting that all gilts should be monitored. In addition, serological data could reveal if the virus is still circulating within the herd via horizontal infections. Vaccination of seronegative gilts and sows is an option that can be considered if removal of carriers alone does not sufficiently control APPV infections.

## Figures and Tables

**Table 1:** Distance matrix from the multiple sequence alignment of six in-farm APPV sequences.

	2013	2015	2016	2017	2018	2019
**2013**		3 aa	7 aa	6 aa	3 aa	5 aa
**2015**	18 nt		4 aa	3 aa	2 aa	3 aa
**2016**	17 nt	15 nt		7 aa	6 aa	6 aa
**2017**	26 nt	18 nt	23 nt		4 aa	1 aa
**2018**	16 nt	13 nt	15 nt	19 nt		5 aa
**2019**	25 nt	21 nt	21 nt	10 nt	20 nt	

## Supplementary Materials

The following are available online at https://www.mdpi.com/1999-4915/12/10/1080/s1, Table S1: List of amplification and sequencing primers used in the study; Table S2: Details on the production data of the studied farm during the 2015–2016 CT outbreak; Table S3: Serum APPV monitoring of gilts selected for breeding at the age of first insemination between January and April in 2016; Table S4: Follow-up of five litters with CT born from gilts for the presence of APPV in serum over time (2015/2016 outbreak); Table S5: Follow-up of boars and gilts until the age of 10 months for the presence of APPV in serum (2015/2016 outbreak); Table S6: Follow-up of boars and gilts until the age of 10 months for the presence of APPV in fecal shed (2015/2016 outbreak); Table S7: APPV E2 sequencing sample list and corresponding APPV viral loads; Table S8: Nucleotide substitutions in the APPV in-farm multiple sequence alignment; Figure S1: Multiple sequence alignment of the partial APPV E2 amino acid sequences chronologically ordered.
