# Supplementary material for: Atypical Porcine Pestivirus Circulation and Molecular Evolution within an Affected Swine Herd
Source: Viruses. 2020 Sep 25;12(10):1080. doi: 10.3390/v12101080 (PMC7599615; doi:10.3390/v12101080)
Supplement: Supplementary file 1 [file viruses-12-01080-s001.pdf]

viruses MDPI

Supplementary Materials: Atypical Porcine Pestivirus Circulation and Molecular Evolution within an Affected Swine Herd

Table S1. List of amplification and sequencing primers used in the study.

Amplification/Sequencing Primers	Primer Sequences
1-F	GGCCTGGGGGTCGGGTCC
12F	TAGACCTTACTGCTTGATAG
12F-W1F	CCAGCGTCACCTTAGTATCG
108-F	ATGGAAAAACAGATTGCA
125F	GAAATGGGTGGACGGAATTG
142F	GTGGACGGAATTGGTGGTAG
923-F	GCTGAAGAATTGGCATATAGC
923F-W1F	ATGGGGTTGACAAGGCGATAAT
1263F	TTATGAGGGACCTGAGTGCG
1488F	GGTTGACAAGGCGATAATACTGA
1488F-P2F	GCAAATGGACTGTGATAACT
1488F-P1R	ATACCTGAGCCAAATAAGTGTT
1773R	GTAAGACTAGAACCCACTTT
1773R-W1R	AGGGATCTATTGATCTGTCT
2742-F	AGGAAAGACGGYTAYTGGCTTCAG
2742F-P2R	TACAATCCACACCGATATCC
2742F-W1F	CATTGACTGGTCTGAGTGATTC
2742F-W1R	GGGAAGCAAGTGGCGGTA
3751-F	AGGGTGACATGTTCCAACTC
3751F-W1F	ATGGTGCTGATGAGGTAGAATA
3968-R	TTGCACTACAGTTGGGAGRC
3968R-W1F	AATCACTCAGACCAGTCAATGT
3968R-P1R-W1R	AGCCTATTATAGCAGTAAGT
3968R-P1R	GGTTGGTCTATGCACATCTT
4822-F	TAGTYCCCTTAAAAGCACCCG
4966-R	CAGCAGAATCCGTAAGTAGC
4966R-W1F	ATGTTCTTGGGATTGTAGGATA
5834-F	CCTGGAAAGAAGTACTCACC
5834F-P1F	TAGGAGATGAGGTCGCTACT
5998-R	GCTTGTGGGTTGACAWCTTTTAG
7048-R	TTGTCGATGTTCATATGGAGC
7048R-P2F	ACCAATATCTGACTATCTAC
7048R-P2F-W1F	GTGGTGCTTCAGCAAGGTAT
7048R-W1F	TTGCCTTGTCTCTCCCAGTCTC
7186F	CACGCA
7186F-P1F	GTGGAATGTAAATGTGGTGCTT
7186F-P1F	ATGGGGACCTAATTTTAATAAC
7186F-P2R	TCTAGTGCCGCCTGATCTAT
7730F	GACGGGAAGAGGTCAATAAGA
7826-F	CCCAAGCCAAGTRGCCATAG
7826F-W1R	GATGTCCACCTCTGCCTGATGA
7963-R	TCTGTGATCTTGGAHCCACG
8680-F	AGGAAGGRGGCGAAGAACC
8680F-P2R	CACTGATCCAGTCACTTAAT
8680F-W1R	CTCTGACCTCGGCTGTTCTC
8680F-W1R-P1F	ATAAAAGAGGGCAAAGTGAAAC
8680F-W1R-P2F	CAGCCATGCCAAAGAATGAG

8680F-W1R-P3F	AAAGAATAGACGTCATAATC
8984-R	CCCTTGGGCCAACTCTTTRG
9306R	CACCTCTCCCTTCTACCATTCCATA
9714-F	CCTGAGGCAGTYACCAGG
9714F-W1R	GTTTGGTGTTTGGACTCTAATG
9734F	AAGTGGGTAAAGCAGAAGCC
9951R-W1F-P1R	GTTTCACTTTGCCCTCTTTTAT
9951R-W1F	CAAGATTGACTGCCTGAA
9951-R	AGTAGTAYTTCTGTATCCGTCCTAC
10459R	AAATGCCACCTGTTTCTCGTA
10840R	AATGACCTGAGCCACTCCTAT
10851R	TCCCACCTACGAATGACCTG
10851R-P1F	GGGGGTAAAGTATGTTGGGGAC
10877R	GTTTCCTTCTGGTCCACTCAA
10881-R	CRAGCTCTTCCCACCTACG
11015-R	TTARTAAGGGGTRAGCATTCTCTGC
11015R-P1R	ATTCTTCTCCTGATCTAGTG

Table S2. Detailed production data of the studied farm during the 2015-2016 CT outbreak[1].

Production Parameters	Q4 2015	Q1 2016	Q2 2016	Q3 2016	Q4 2016
Total litters born	316	289	437	336	308
Total live born piglets	4223	4315	5817	4571	4333
Total weaned piglets	3615	3236	5105	3955	3543
Total sold piglets incl. runts	3553	2718	4993	2876	3422
Weaned/litter	11.4	10.9	11.7	11.8	11.5
Sold/litter	11.24	9.1	11.4	11.5	11.1
% Mortality in farrowing unit	16.8	33.3	13.9	15.6	22.2
% Mortality in nursery unit	1.7	19	2.2	2	3.5

[1] Piglets were vaccinated with a Porcine Reproductive and Respiratory Syndrome Virus (PRRSV) live vaccine at 2 weeks of age, and with a combined Porcine Circovirus type 2 (PCV2) and Mycoplasma hyopneumoniae (M. hyo) vaccine at 3 weeks of age. PRRSV was regularly diagnosed in the farm and therefore a vaccination strategy in sows and piglets was set up. There were no other pathogens diagnosed in regular screenings.

Table S3. Serum APPV monitoring of gilts selected for breeding at the age of first insemination between January and April in 2016.

Date	Number of Gilts	Number of Positive Gilts	Average Copies/mL
28-jan-2016	25	2	2.99×10^2
24-feb-2016	31	6	1.68×10^4
11-mar-2016	52	18	2.66×10^4
1-apr-2016	41	1	6.31×10^4
19-apr-2016	47	3	2.28×10^3
Total	196	30	-

Table S4. Follow up of five litters with CT born from gilts for the presence of APPV in serum over time (2015/2016 outbreak).

	5 weeks		8 weeks		18 weeks	
	Count	Percentage	Count	Percentage	Count	Percentage
Litter 1	8/9	89%	n/a	n/a	4/5	80%
Litter 2	10/10	100%	10/10	100%	3/10	30%
Litter 3	10/10	100%	10/10	100%	1/4	25%
Litter 4	9/9	100%	9/9	100%	3/6	50%
Litter 5	12/12	100%	n/a	n/a	4/8	50%
Overall	49/50	98%	27/27	100%	15/30	45%
Average APPV copies/mL	3.77×10^5		6.95×10^5		3.86×10^3	

Table S5. Follow up of boars and gilts until the age of 10 months for the presence of APPV in serum (2015/2016 outbreak).

Animal	Group	APPV in Serum (copies/mL)									
		**5w**	**8w**	**18w**	**22w**	**24w**	**28w**	**32w**	**36w**	**40w**	**44w**
Gilt 87	1	6.75×10^4	1.77×10^6	1.17×10^3	Neg	Neg	Neg	Neg	Neg	Neg	Neg
Gilt 100	1	7.05×10^4	5.68×10^5	8.95×10^3	1.61×10^2	Neg	Neg	Neg	Neg	Neg	Neg
Gilt 267[1]	1	Neg	Neg	Neg	Neg	Neg	Neg	Neg	Neg	Neg	Neg
Gilt 92	2	1.64×10^4	2.19×10^5	Neg	Neg	Neg	Neg	Neg	Neg	Neg	Neg
Gilt 93	2	1.68×10^4	4.74×10^4	Neg	Neg	Neg	Neg	Neg	Neg	Neg	Neg
Gilt 270[1]	2	Neg	Neg	Neg	1.50×10^2	Neg	Neg	Neg	Neg	Neg	Neg
Boar 152	3	4.37×10^4	n/t[2]	4.65×10^4	Neg	Neg	Neg	1.52×10^3	Neg	Neg	Neg
Boar 277[1]	3	Neg	Neg	Neg	Neg	Neg	Neg	Neg	Neg	Neg	Neg

[1] APPV negative animals; [2] Not tested.

Table S6. Follow up of boars and gilts until the age of 10 months for the presence of APPV in fecal shed (2015/2016 outbreak).

Animal	Group	APPV in Serum [1]												
		**22w**	**24w**	**28w**	**32w**	**36w**	**37w**	**38w**	**39w**	**40w**	**41w**	**42w**	**43w**	**44w**
Gilt 87	1	Pos	Neg	Pos	Pos	Pos	Pos	Pos	Neg	Pos	Pos	Pos	Pos	Pos
Gilt 100	1	Pos	Neg	Pos	Neg	Neg	Pos	Neg	Pos	Neg	Neg	Neg	Neg	Neg
Gilt 267 [2]	1	Neg	Neg	Neg	Neg	Neg	Neg	Neg	Neg	Neg	Neg	Neg	Neg	Neg
Gilt 92	2	Neg	Neg	Pos	Neg	Pos	Neg	Neg	Neg	Pos	Neg	Neg	Neg	Neg
Gilt 93	2	Neg	Pos	Pos	Pos	Pos	Neg	Neg	Neg	Neg	Pos	Neg	Pos	Neg
Gilt 270 [2]	2	Neg	Neg	Pos	Neg	Pos	Pos	Neg	Neg	Neg	Neg	Neg	Neg	Neg
Boar 152	3	Pos	Pos	Pos	Pos	Pos	Pos	Pos	Pos	Pos	Pos	Pos	Pos	Pos
Boar 277 [2]	3	Pos	Neg	Neg	Neg	Pos	Neg	Neg	Neg	Neg	Neg	Neg	Neg	Neg

[1] No quantitative analyses due to unknown dilution factor; [2] APPV negative animal

Table S7. APPV E2 sequencing sample list and correspondent APPV viral loads.

Date	Number of positive pigs	Number of sequenced pigs	Tag number	APPV copies/mL
dec-2013	-	1	9464[1]	2.77×10^2
nov-2015	-	2	42039[1]	4.99×10^2
			46361[1]	4.16×10^4
28-apr-2016	12	8	39358	2.58×10^3
			39360	8.38×10^3
			39361	7.62×10^3
			39362	1.81×10^4
			39364	8.52×10^3
			39366	8.07×10^3
			39367	7.91×10^3
			39368	1.38×10^4
18-may-2016	6	6	6815	8.17×10^2
			6816	6.47×10^2
			6817	6.17×10^2
			6818	5.77×10^2
			6819	5.81×10^2
			6813[1]	4.21×10^5
10-jun-2016	16	6	6844	5.77×10^2
			6847	4.99×10^2
			6848	5.81×10^2
			6849	4.59×10^2
			6850	3.73×10^2
			6851	5.36×10^2
13-jul-2016	2	0	n/a	n/a
10-may-2017	9	8	6820-1	9.67×10^3
			6820-2	1.02×10^4
			6820-3	4.38×10^4
			6827-1	1.47×10^3
			6827-2	1.09×10^3
			6827-3	7.41×10^2
			6837-1	1.01×10^3
			6837-2	9.43×10^2
23-nov-2017	14	4	7450	2.83×10^3
			7451	2.69×10^3
			7452[1]	2.05×10^6
			7453	4.34×10^3
8-jan-2018	5	1	7501	7.97×10^2
16-feb-2018	19	3	7589	1.73×10^2
			7591[1]	2.58×10^4
			7592	1.25×10^2
18-apr-2018	7	2	7667	3.21×10^2
			7671	6.38×10^2
28-mar-2019	4	4	7981[1]	2.01×10^5
			7982	2.99×10^2
			7983	3.37×10^2
			7985	6.09×10^2
17-apr-2017	5	1	7998	4.64×10^2
1-nov-2019	4	3	8246	2.51×10^2
			8247	1.54×10^1
			8248	2.56×10^2
2-dec-2019	5	2	8262	6.08×10^1
			8264	7.90×10^3
Total	108	51		-

[1] Full genome sequence was obtained from these samples.

Table S8. Nucleotide substitutions in the APPV in-farm multiple sequence alignment.

Prot [1]	Pos [1]	Codon 1 [2]	AA 1 [2]	Years [3]	Condon 2 [2]	AA 2 [2]	Years [3]	Subs [4]
Npro	19	ACG	Thr	2013, 2015, 2016, 2019	ACT	Thr	2017	S
	23	GTA	Val	2013, 2015, 2016, 2017	GTG	Val	2019	S
	48	TCG	Ser	2013	CCG	Pro	2015, 2016, 2017, 2018, 2019	NS
	104	CTG	Leu	2013, 2016, 2018	CTA	Leu	2015, 2017, 2019	S
	132	AGG	Arg	2013, 2015, 2016, 2018, 2019	AGA	Arg	2017	S
	152	CAC	His	2013, 2015, 2017, 2018, 2019	CTC	Leu	2016	NS
C	269	GTT	Val	2013, 2015, 2016, 2017, 2018	GTC	Val	2019	S
	285	ATC	Ile	2013	ATT	Ile	2015, 2016, 2017, 2018, 2019	S
Erns	307	ATT	Ile	2013, 2015, 2016, 2017	ATC	Ile	2019	S
	418	GAC	Asp	2013, 2015[5], 2016, 2017, 2019	GAT	Asp	2015	S
	442	TCG	Ser	2013, 2015, 2017, 2018, 2019	TCA	Ser	2016	S
E1	563	GTC	Val	2013, 2015, 2016, 2017, 2019	GCC	Ala	2018	NS
	567	GTC	Val	2013	GTT	Val	2015, 2016, 2017, 2018, 2019	S
E2	726	ATA	Ile	2013, 2015, 2016, 2017, 2018	GTA	Val	2019	NS
	752	GAT	Asp	2013, 2015, 2016, 2018	GGT	Gly	2017, 2019	NS
	810	CTG	Leu	2013	CTA	Leu	2015, 2016, 2017, 2018, 2019	S
	881	GAT	Asp	2013, 2015, 2016	GAC	Asp	2017, 2019	S
p7	no substitutions found in 2013, 2015, 2016, 2017, 2018, 2019							
NS2	1054	ACT	Thr	2013, 2015, 2016, 2018, 2019	ACC	Thr	2017	S
	1122	AGA	Arg	2013, 2018	AAA	Lys	2015, 2016, 2017, 2019	NS
	1161	TTA	Leu	2013, 2015, 2017, 2018	TTG	Leu	2016, 2019	S
	1198	AGC	Ser	2013, 2015, 2016, 2017, 2019	AGT	Ser	2018	S
NS3	1392	AAG	Lys	2013, 2015, 2016, 2018	AAA	Lys	2017, 2019	S
	1814	AAA	Lys	2013, 2015, 2016, 2017, 2018	AAG	Lys	2019	S
	1855	CAA	Gln	2013, 2016	CAG	Gln	2015, 2017, 2018, 2019	S
NS4A	no substitutions found in 2013, 2015, 2016, 2017, 2018, 2019							
NS4B	2241	GCC	Ala	2013, 2015, 2016, 2018	GCT	Ala	2017, 2019	S
	2249	ACC	Thr	2013, 2015, 2016, 2017, 2018	ACA	Thr	2019	S

	2383	TCT	Ser	2013	TCC	Ser	2015, 2016, 2017	S
**NS5A**	2437	AAG	Lys	2013, 2015, 2016, 2018	AGG	Arg	2017, 2019	NS
	2550	GGG	Gly	2013, 2015, 2017, 2018	GAG	Glu	2016	NS
	2560	GAC	Ala	2013, 2015, 2016, 2017	GCC	Ala	2018	S
	2652	GTA	Val	2013	ATA	Ile	2015, 2016, 2017, 2018	S
	2676	AGC	Ser	2013	AGT	Ser	2015, 2016, 2017, 2018	S
	2680	CTA	Leu	2013, 2016	TTA	Leu	2015, 2017, 2018	S
	2693	GAA	Glu	2013, 2015, 2016, 2017	GAG	Glu	2018	S
	2741	TAT	Tyr	2013, 2015, 2016, 2017	TAC	Tyr	2018	S
	2766	CCC	Pro	2013	CCT	Pro	2015, 2016, 2017	S
	2824	GCG	Ala	2013, 2015, 2016	ACG	Thr	2017	NS
	2848	ACA	Thr	2013, 2015, 2017	ACG	Thr	2016	S
**NS5B**	2905	ACA	Thr	2013, 2015[5], 2016, 2017, 2018, 2019	ACG	Thr	2015	S
	2979	GAC	Asp	2013, 2015, 2017, 2018, 2019	AAC	Asn	2016	NS
	3144	TCA	Ser	2013	TCG	Ser	2015, 2016, 2017, 2018, 2019	S
	3166	AAC	Asn	2013, 2015, 2017, 2018, 2019	ACC	Thr	2016	NS
	3171	GAA	Glu	2013, 2015, 2016, 2018	GAG	Glu	2017, 2019	S
	3243	AGT	Ser	2013, 2015, 2016, 2018	AGC	Ser	2017, 2019	S
	3259	ACC	Thr	2013, 2015, 2016, 2018	ACT	Thr	2017, 2019	S

[1] Substitutions are classified in the corresponding viral proteins (Prot) and the codon position (Pos) in the complete coding sequence. [2] Codon 1 and amino acid 1 (AA 1) correspond to the first appearance among the sequences, while codon 2 and amino acid 2 (AA 2) correspond to the variants found later in time. [3] When a year is not presented in the table means that the sequence of that region is not available for the specific year. Data missing from 2018 fragments 1–92 bp (Npro) and 8274–8634 bp (NS5A); from 2019 fragment 7463–8628 bp (NS5A); last 12 bp of the CDS (10894–10905 bp) from 2013, 2014, 2015, 2017, 2018 and 2019. [4] S: synonymous substitution; NS: non-synonymous substitution, highlighted in blue. [5] Sequence from 2015 obtained through Illumina sequencing included in the analysis. There are 2 mismatches (synonymous) found between Sanger Sequencing (GenScript) and Illumina sequencing (UMC Amsterdam AMC) from 2015 samples.

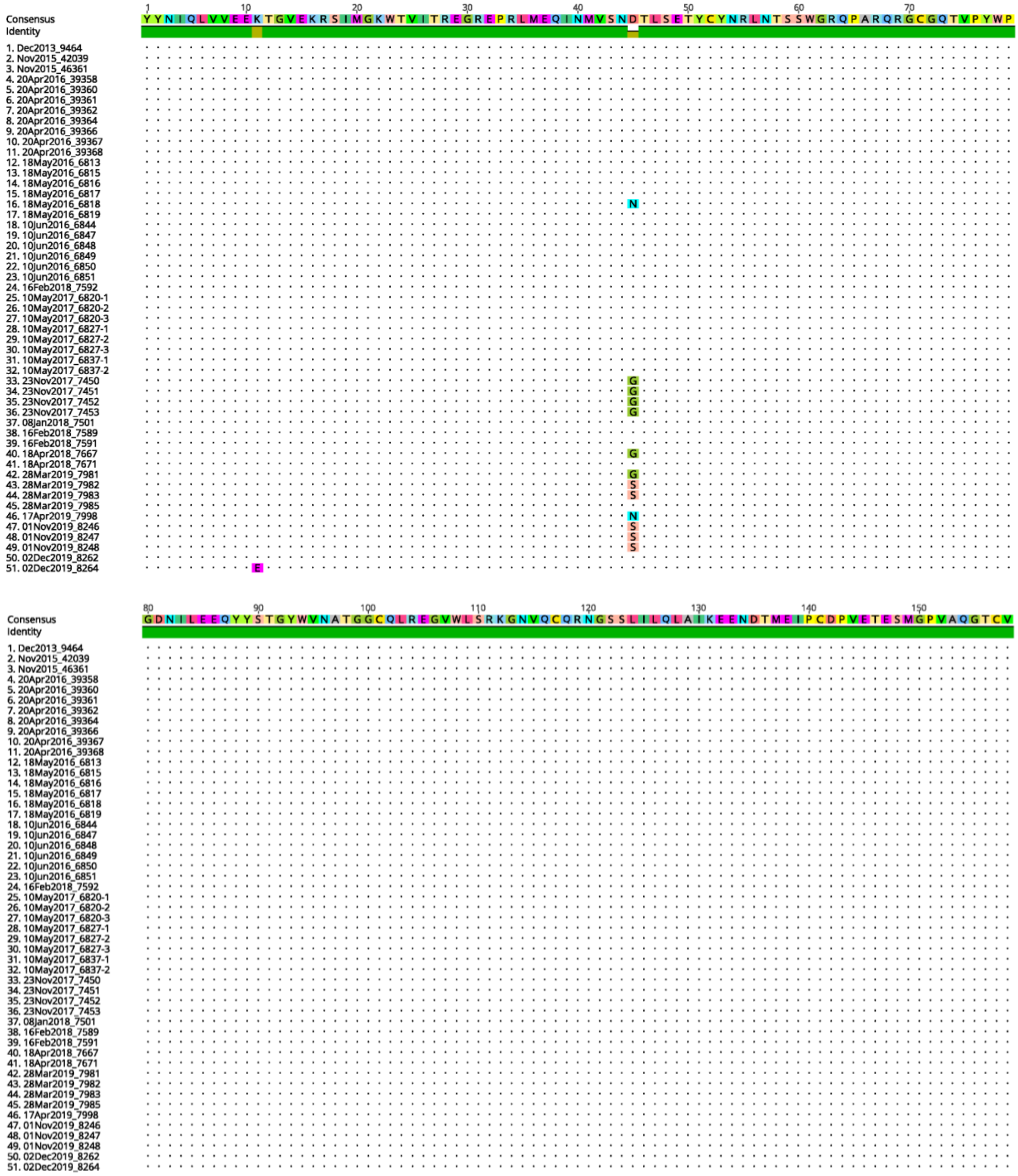
1 10 20 30 40 50 60 70
Consensus
YYNIQLVVEEKTGVEKRSIMGKWTVITREGREPRLMEQINMVSNDTLSETYCYNRLNTSSWGRQPARQRGCGQTVPYWP
Identity
1. Dec2013_9464
2. Nov2015_42039
3. Nov2015_46361
4. 20Apr2016_39358
5. 20Apr2016_39360
6. 20Apr2016_39361
7. 20Apr2016_39362
8. 20Apr2016_39364
9. 20Apr2016_39366
10. 20Apr2016_39367
11. 20Apr2016_39368
12. 18May2016_6813
13. 18May2016_6815
14. 18May2016_6816
15. 18May2016_6817
16. 18May2016_6818
17. 18May2016_6819
18. 10Jun2016_6844
19. 10Jun2016_6847
20. 10Jun2016_6848
21. 10Jun2016_6849
22. 10Jun2016_6850
23. 10Jun2016_6851
24. 16Feb2018_7592
25. 10May2017_6820-1
26. 10May2017_6820-2
27. 10May2017_6820-3
28. 10May2017_6827-1
29. 10May2017_6827-2
30. 10May2017_6827-3
31. 10May2017_6837-1
32. 10May2017_6837-2
33. 23Nov2017_7450
34. 23Nov2017_7451
35. 23Nov2017_7452
36. 23Nov2017_7453
37. 08Jan2018_7501
38. 16Feb2018_7589
39. 16Feb2018_7591
40. 18Apr2018_7667
41. 18Apr2018_7671
42. 28Mar2019_7981
43. 28Mar2019_7982
44. 28Mar2019_7983
45. 28Mar2019_7985
46. 17Apr2019_7998
47. 01Nov2019_8246
48. 01Nov2019_8247
49. 01Nov2019_8248
50. 02Dec2019_8262
51. 02Dec2019_8264
80 90 100 110 120 130 140 150
Consensus
GDNILEEQYYSTGYWVNATGGCQLREGVWLSRKGNVQCQRNGSSLILQLAIKEENDTMEIPCDPVETESMGPVAQGTCV
Identity
1. Dec2013_9464
2. Nov2015_42039
3. Nov2015_46361
4. 20Apr2016_39358
5. 20Apr2016_39360
6. 20Apr2016_39361
7. 20Apr2016_39362
8. 20Apr2016_39364
9. 20Apr2016_39366
10. 20Apr2016_39367
11. 20Apr2016_39368
12. 18May2016_6813
13. 18May2016_6815
14. 18May2016_6816
15. 18May2016_6817
16. 18May2016_6818
17. 18May2016_6819
18. 10Jun2016_6844
19. 10Jun2016_6847
20. 10Jun2016_6848
21. 10Jun2016_6849
22. 10Jun2016_6850
23. 10Jun2016_6851
24. 16Feb2018_7592
25. 10May2017_6820-1
26. 10May2017_6820-2
27. 10May2017_6820-3
28. 10May2017_6827-1
29. 10May2017_6827-2
30. 10May2017_6827-3
31. 10May2017_6837-1
32. 10May2017_6837-2
33. 23Nov2017_7450
34. 23Nov2017_7451
35. 23Nov2017_7452
36. 23Nov2017_7453
37. 08Jan2018_7501
38. 16Feb2018_7589
39. 16Feb2018_7591
40. 18Apr2018_7667
41. 18Apr2018_7671
42. 28Mar2019_7981
43. 28Mar2019_7982
44. 28Mar2019_7983
45. 28Mar2019_7985
46. 17Apr2019_7998
47. 01Nov2019_8246
48. 01Nov2019_8247
49. 01Nov2019_8248
50. 02Dec2019_8262
51. 02Dec2019_8264

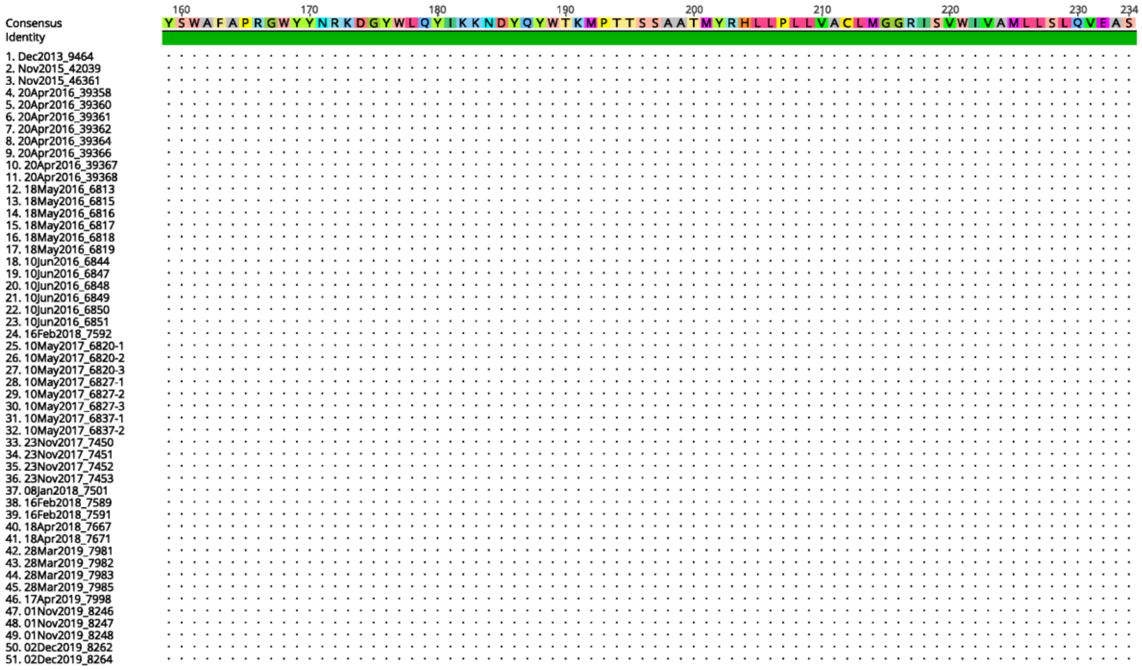

Figure S1. Multiple sequence alignment of the partial APPV E2 amino acid sequences chronologically ordered.
